# Commentary: Traditional Chinese exercises on pain and disability in middle-aged and elderly patients with neck pain: a systematic review and meta-analysis of randomized controlled trials

**DOI:** 10.3389/fnagi.2023.1158900

**Published:** 2023-07-25

**Authors:** Zhejia Cao, Zhiliang Li, Qiqi Lu

**Affiliations:** ^1^The Second School of Clinical Medicine, Southern Medical University, Guangzhou, China; ^2^The First School of Clinical Medical, Guangdong Medical University, Zhanjiang, China

**Keywords:** traditional Chinese exercises, neck pain, disability, meta-analysis, complementary and alternative therapy

## 1. Introduction

An article by Kong et al. (2022) titled “*Traditional Chinese Exercises on Pain and Disability in Middle-Aged and Elderly Patients with Neck Pain: A Systematic Review and Meta-Analysis of Randomized Controlled Trials*” caught our attention.

Six electronic databases were searched to identify 21 randomized controlled studies, this study aimed to examine the effects of traditional Chinese exercise (TCEs) on neck pain and disability in middle-aged and elderly individuals. Outcome data included Pain, range of motion, disability, and so on. The results of the final meta-analysis showed that TCEs showed positive complementary effects in relieving pain, especially Baduanjin exercises. The Baduanjin exercises also improved flexion and extension of the neck simultaneously. In addition, the aggregated results indicated that TCEs alone showed beneficial effects in improving disability and relieving pain compared with a waiting list. The authors conclude that there was positive evidence to support the clinical use of TCEs, as a complementary therapy, for middle-aged and elderly patients with neck pain, especially Baduanjin exercises.

This is undeniably an excellent article, and the evidence of TCEs for neck pain maintains controversial, it is very meaningful to break geographical restrictions in clinical practice and recommend traditional Chinese exercises to middle-aged and elderly patients with neck pain around the world. However, this article raised some concerns for us.

## 2. Insufficient inclusion of studies

The Methodology stated that the literature search was conducted on January 2022. The exact date of retrieval is not mentioned in the original text. We found that the article omitted several Chinese RCTS related to the Baduanjin movement, such as a study involving 64 people published in 2019 by Pengcheng et al. (2019). And in a systematic review of Baduanjin in the treatment of neck pain in middle-aged and elderly adults by Liu et al. (2022) at least two more RCTS were included than in the paper by Kong et al. (2022). The missing RCTS were all published before January 2022. TCEs included Baduanjin, Yijinjing, Tai Chi, Qigong, and Five-animal exercises, just the Baduanjin movement missed many RCTS, other Yi Jin Jing, Tai Chi? It may have been due to an improper search strategy used in the meta-analysis. The authors concluded that Baduanjin may be the most beneficial TCEs, but the relevant RCTS of Baduanjin were omitted. Will this affect the results? Multiple types of interventions require rigorous and complex meta-analysis search strategies.

## 3. Data classification extraction error

In Table 1, Huang et al. (2020), study showed that the intervention of the experimental group in the study was Baduanjin. However, in the original paper of Huang et al., the intervention group was Yijinjing. These are two very different interventions, therefore, the baseline data for the intervention group in Table 1 is incorrect.

In the original, the change of the experimental group was 2.47 ± 0.44 and that of the control group was 1.88 ± 0.30. However, in the study of Kong et al. the data extracted from the intervention group in Figure 2 was 2.47 ± 0.47 and that of the control group was 1.87 ± 0.39 (in Figure 2A of the forest plot, subgroup analysis of Yijinjing, about Huang et al.'s study in 2020). This is different from the data given in the original article.

## 4. Forest plot contains effects model error and error of interpretation

In Figure 2C, the analysis model should use the random effects model rather than the fixed effects model.

In Figure 2A, as for the interpretation of pain, the subgroup analysis results of the forest map showed that Baduanjin, Yijinjing, and other traditional exercises were effective (*P* < 0.05). However, the test for subgroups differences showed that the *P* value was 0.90 and *I*^2^ was 0% (Figure 2A), indicating that there was no significant difference in the analgesic effect of the three subgroups. Therefore, the statement “especially Baduanjin exercises....” in the original text is completely wrong, the available evidence does not say which intervention is the most effective.

We respectfully appreciate that Kong et al. provided us with an important meta-analysis focusing on Traditional Chinese Exercises on Pain and Disability in Middle-Aged and Elderly Patients with Neck Pain. The author of this review hopes that these reviews will improve the article.

## Author contributions

All authors listed have made a substantial, direct, and intellectual contribution to the work and approved it for publication.
